# Negative fat talk and sleep quality among Chinese college students: associations involving wellbeing and loneliness

**DOI:** 10.3389/fpsyg.2026.1969448

**Published:** 2026-10-23

**Authors:** Mingshou Zhang, Zidie Zang, Guifang Su, Lanqi’er Li, Yiqiao Chen, Zhimin Yi, Ming Hao

**Affiliations:** School of Public Health and Health Management, Gannan Medical University, Ganzhou City, China

**Keywords:** body image, loneliness, negative fat talk, poor sleep quality, sleep health, university students, wellbeing

## Abstract

**Background:**

This study examined the relationship between negative fat talk and poor sleep quality in Chinese college students, and tested the roles of wellbeing and loneliness in this association.

**Methods:**

A cross-sectional survey included 2,056 students in Jiangxi Province, China. Negative fat talk, wellbeing, loneliness, physical activity, and sleep quality were assessed with the BTS, WHO-5, ULS-8, PARS-3, and PSQI, respectively. The additional observed-score analyses included 1,970 participants with observed PSQI total scores. The original latent-variable parallel mediation model was retained; a fully adjusted observed-score model was added.

**Results:**

The original classification recorded 984 students (47.9% of the study sample) with poor sleep quality. In the original sex-adjusted regression, negative fat talk was associated with higher PSQI scores (standardized *β* = 0.060, *p* = 0.003). In the additional fully adjusted regression, its unstandardized coefficient was 0.046 (95%CI 0.016–0.076; *p* = 0.003). The original structural model reported an overall indirect association of 0.223, with specific associations of 0.118 through wellbeing and 0.105 through loneliness.

**Conclusion:**

Negative fat talk was associated with poorer self-reported sleep quality. The parallel indirect paths describe cross-sectional statistical associations.

## 1 Introduction

Sleep quality is an important indicator of physical and mental health of college students. Past system review and meta-analysis show that the incidence of sleep problems in college students is relatively high, mainly manifested as difficulty in falling asleep, insufficient sleep time, decreased sleep efficiency and impaired daytime function (Li et al., 2018; Seoane et al., 2020). Sleep is not only a physiological rest process, but also closely related to emotional recovery, cognitive integration and the mental health maintenance (Palmer et al., 2024; Scott et al., 2021). In view of the high prevalence of sleep problems and its impact on the functional state, poor sleep quality has become an important concern in the field of sleep health of college students.

In recent years, issues related to appearance, body type, and body image have received more and more attention among college students. Body image research shows that how individuals’ perception and evaluation of their own bodies are not only affected by personal cognition and subjective evaluation, but also shaped by peer relationships, media environments and broader social and cultural norms (Cash, 2004; Rodgers et al., 2014). In the social media environments, idealized body displays and comparison-oriented information browsing further strengthen appearance pressure and self-examination (Brown and Tiggemann, 2016; Fardouly et al., 2015). Against this background, the body-related expression in daily peer communication provides an observable entry point for understanding the connection between appearance stress and sleep health of college students.

Negative fat talk refers to negative statements about one’s own weight or body shape, such as a perceived need to lose weight (Lin et al., 2021; Mills and Fuller-Tyszkiewicz, 2016). Social comparison theory describes how evaluations of the self can arise in relation to other people (Festinger, 1954), while objectification theory links external evaluation of appearance to body monitoring (Fredrickson and Roberts, 1997). Previous studies have found that negative fat talk may be a maladjusted body-related self-expression, which is related to self-monitoring, appearance internalization and emotional vulnerability, and is closely related to physical dissatisfaction, perceived stress and peer interaction norms (Barbeau et al., 2022; Warren et al., 2012). Similar phenomena also exist in the group of male college students, indicating that this phenomenon is not limited to women’s daily communication situations (Engeln et al., 2013).

Under the broader risk framework, weight mockery, physical dissatisfaction and negative body talk often occur with restrictive diet, depressive symptoms and other mental health problems (Christensen Pacella and Forbush, 2024; Hao et al., 2023; Hooper et al., 2021; Richard et al., 2016; Yang et al., 2022). Therefore, negative fat talk can be regarded as an observable form of body-related negative self-expression, which not only reflects the individual’s appearance concerns, but also suggests that he faces a broader social and psychosocial pressure. Continuous body-related self-evaluation may also further lead to long-term emotional burden, maladaptive self-focus cognition, and increase susceptibility to sleep-related problems.

Existing studies suggest that body dissatisfaction and weight-related negative experiences may extend into the domain of sleep health. Research among university students has shown that body dissatisfaction is significantly associated with poorer sleep quality, exercise intensity, and depression (Hao et al., 2023), whereas another study found that weight bias internalization was positively associated with insomnia symptom severity (Christensen Pacella and Forbush, 2024). Among adolescents, dissatisfaction with height and weight has also shown both concurrent and longitudinal associations with sleep disturbance (Lin et al., 2025). Together, these findings suggest that negative body evaluation may be linked to sleep health through cognitive and emotional processes such as rumination, vigilance, and presleep arousal. Negative fat talk may also reinforce repetitive self-focused cognition, shame-related rumination, and heightened sensitivity to external evaluation, all of which are associated with presleep cognitive arousal and sleep disturbance.

Among the potential mediating mechanisms, wellbeing and loneliness deserve particular attention. As important indicators of positive psychological resources, subjective wellbeing and positive affect are consistently associated with sleep quality; better sleep is often accompanied by more positive affect and better day-to-day recovery (Bacaro et al., 2024; Ong et al., 2017). Wellbeing may represent a positive psychological resource through which negative fat talk is related to sleep quality. Students who often talk about negative fat may have fewer positive experiences and feel a lower level of psychological recovery, which is further associated with poor sleep quality.

In contrast, loneliness reflects an individual’s subjective perception of the insufficient quantity or quality of their social connections. Previous reviews and studies of university student samples have demonstrated stable associations of loneliness with poorer sleep quality, bedtime procrastination, and emotional distress (Deng et al., 2023; Ostrow et al., 2025; Parlikar et al., 2026; Wang et al., 2025). Loneliness is also relatively common in university populations and is shaped by social-ecological contexts and interpersonal support (Akram et al., 2025; Wenig et al., 2025). Loneliness may therefore represent a social-psychological pathway linking negative body-related expression with poorer sleep quality. Research among university students also links loneliness to psychological and subjective wellbeing (Çiçek, 2021). In the present study, wellbeing and loneliness were assessed as correlated but distinct psychological correlates of sleep quality.

From a mechanistic perspective, social connectedness and personal agency exert a sustained influence on loneliness, and loneliness in turn undermines health outcomes (Ybarra and Chan, 2025; Zheng et al., 2026). This suggests that if negative fat talk is accompanied by fear of being evaluated, shame, and social withdrawal, it may weaken one’s sense of connectedness and thereby enter the process through which sleep problems develop. Although sleep, wellbeing, and loneliness have been widely examined among college students, negative fat talk has rarely been studied as a body-related psychosocial factor of sleep health in university populations. Body image research, including sociocultural models and studies of appearance internalization, suggests that negative body-related expression may serve as an antecedent cue for subsequent psychological and behavioral outcomes (Kidd et al., 2024; Schaefer et al., 2015). Against this background, this study examined the association between negative fat talk and poor sleep quality among Chinese college students and tested the roles of wellbeing and loneliness in this association.

We examined the hypothesized parallel associations of negative fat talk with PSQI scores through wellbeing and loneliness. Because all study variables were measured cross-sectionally, the indirect paths are statistical decompositions rather than evidence that one construct preceded another. An exploratory comparison assessed the difference between the two specific indirect associations in an adjusted observed-score model.

*H1*: Higher negative fat talk would be associated with poorer sleep quality among Chinese college students.

*H2*: Lower wellbeing would statistically mediate the association between negative fat talk and poorer sleep quality.

*H3*: Higher loneliness would statistically mediate the association between negative fat talk and poorer sleep quality.

*Exploratory H4*: The magnitudes of the two specific indirect associations may differ; their contrast is evaluated with a bootstrap confidence interval.

## 2 Materials and methods

### 2.1 Study design and participants

This observational cross-sectional study was conducted from October 2024 to July 2025 at three universities in Ganzhou and one university in Ji’an, Jiangxi Province, China, using convenience sampling. The study was reported in accordance with the STROBE guidelines for cross-sectional studies. Data were collected either by distributing questionnaire QR codes during evening self-study classes or by asking teachers to forward the QR code to students. An electronic informed consent form was presented on the first page of the questionnaire, and students who did not consent could not proceed.

The inclusion criteria were full-time student status in one of the participating universities, age 18–29 years, provision of informed consent, and absence of serious physical illness. The exclusion criteria were failure to provide informed consent, missing basic information, clearly invalid responses, and age outside the specified range. In the end, a total of 2,056 participants were included in the analysis. Sample size was estimated using G*Power 3.1.9.7 (Faul et al., 2009). Assuming 90% power, a significance level of 0.05, and four predictors, at least 776 participants were required to detect a small effect size in multiple linear regression. The actual sample size exceeded this requirement.

The study has been approved by the Ethics Committee of Gannan Medical University (approval number: 2021110). Electronic informed consent was obtained before participation.

### 2.2 Measures

The questionnaire captured sex, age, grade, major, school, only-child status, place of origin, and monthly living expenses. As described in the final thesis, height was measured using a Seca 213 portable stadiometer (resolution 0.1 cm), and weight using a Tanita BC610 body composition analyzer (resolution 0.1 kg). BMI was calculated as weight in kilograms divided by squared height in meters. The archived dataset does not contain individual measurement or calibration logs; the instruments and their precision reflect the documented field procedure. BMI categories followed the Chinese adult standard WS/T 428–2013: <18.5, 18.5–<24.0, 24.0–<28.0, and≥28.0 kg/m^2^ (National Health and Family Planning Commission of the PRC, 2013).

Physical activity was measured with the Physical Activity Rating Scale-3 (Yuan and You, 2022). Negative fat talk was assessed with five items from the Body Talk Scale (BTS), including an item about needing to lose weight; the score range was 5–30 (Wu et al., 2024). The WHO-5 measured wellbeing with five items, including an item about feeling cheerful (range 0–25) (WHO-5) (Topp et al., 2015). The ULS-8 assessed loneliness with eight items, including an item about lacking companionship; two positively worded items were reverse scored (range 8–32) (Hays and DiMatteo, 1987). Sleep quality was evaluated with the PSQI, which sums seven components covering subjective quality, latency, duration, efficiency, disturbances, sleep medication, and daytime dysfunction (range 0–21) (Buysse et al., 1989). A total PSQI score >5 indicated poor sleep quality. Higher scores indicated more physical activity, negative fat talk, wellbeing, loneliness, and poorer sleep quality, respectively.

Internal consistency was acceptable to excellent, with Cronbach’s α coefficients of 0.847 for negative fat talk, 0.924 for wellbeing, 0.801 for loneliness, and 0.886 for sleep quality.

### 2.3 Statistical analysis

The original analyses used SPSS 26.0 and Mplus 8.3. Descriptive statistics were reported as means and standard deviations or counts and percentages. The original regression and latent mediation results were retained. The revised analyses addressed the reviewer requests for expanded covariate adjustment, a comparison of indirect paths, and a shared-method diagnostic.

Spearman rank correlations were used for the main variables because the PARS-3 score was right-skewed. Original unadjusted and sex-adjusted regression results are retained in Table 1. An additional ordinary least squares model adjusted for sex, age, BMI, physical activity, grade, major, only-child status, place of origin, monthly expenses, and standardized school group (Table 2). HC3 robust standard errors supplied its 95% confidence intervals. Unambiguous school-name spelling variants were pooled, with seven uncertain entries grouped separately. A sensitivity model omitted four PSQI-complete records with implausible BMI (<12 kg/m^2^) or PARS-3 scores above the stated maximum of 100.

**Table 1:** Multiple linear regression analysis for sleep quality.

Model	Predictor	*t*	*β*	VIF	*p*
Unadjusted	Physical activity	0.574	0.011	1.015	0.566
	Negative fat talk	3.164	0.063	1.107	0.002
	wellbeing	−16.144	−0.354	1.346	<0.001
	Loneliness	11.598	0.252	1.325	<0.001
Adjusted for sex	Physical activity	1.034	0.021	1.122	0.301
	Negative fat talk	2.978	0.06	1.12	0.003
	wellbeing	−16.024	−0.352	1.351	<0.001
	Loneliness	11.676	0.254	1.33	<0.001

**Table 2:** Expanded-covariate regression for observed PSQI total (*n* = 1970).

Predictor	*B*	95% HC3 CI	Standardized *β*	*p*
Physical activity	0.003	−0.006 to 0.013	0.017	0.483
Negative fat talk	0.046	0.016 to 0.076	0.076	0.003
wellbeing	−0.214	−0.244 to −0.184	−0.351	<0.001
Loneliness	0.199	0.163 to 0.235	0.255	<0.001

Model *R*^2^ = 0.316. Additional covariates: sex, age, BMI, grade, major, only-child status, origin, monthly expenses, and standardized school group. Physical activity was included among predictors. HC3 robust confidence intervals are reported.

Supplementary measurement checks were conducted for negative fat talk, wellbeing, loneliness, and sleep quality. Model adequacy was judged using CFI, TLI, RMSEA, and SRMR (Hu and Bentler, 1999). The original Mplus parallel-mediation model used latent wellbeing and loneliness constructs and applied bias-corrected bootstrapping with 1,000 resamples. Table 3 presents the effect estimates, standard errors, and *p* values reported in Table 2 of the final thesis. Its original estimates were retained because age was outside that model and the original model variables were unchanged. An additional fully adjusted observed-score sensitivity analysis used the same covariates as Table 2 and 1,000 percentile-bootstrap resamples. A 25-indicator unrotated principal-component diagnostic assessed shared-method variance among 1,956 indicator-complete records. Indirect associations were interpreted as cross-sectional statistical decompositions.

**Table 3:** Direct, indirect, and total effects in the original latent-variable mediation model.

Effect type	Path	Estimate	SE	*p*	95% bootstrap CI
Direct effect	Negative fat talk → Sleep quality	0.057	0.029	0.05	0.008–0.100
Total indirect effect	Negative fat talk → Sleep quality	0.223	0.017	<0.001	0.194–0.251
Indirect effect	Negative fat talk → Loneliness → Sleep quality	0.105	0.012	<0.001	0.086–0.125
Indirect effect	Negative fat talk → wellbeing → Sleep quality	0.118	0.014	<0.001	0.096–0.139
Total effect	Negative fat talk → Sleep quality	0.280	0.029	<0.001	0.225–0.323

## 3 Results

### 3.1 Sample characteristics

Sample characteristics are presented in Table 4.

**Table 4:** Sample characteristics.

Variable	Category	*n*	%
Sex	Male	1,068	51.9
	Female	988	48.1
Major	Liberal arts	226	11.0
	Science	591	28.8
	Engineering	492	23.9
	Medicine	656	31.9
	Arts or sports	91	4.4
Only child	Yes	191	9.3
	No	1865	90.7
Place of origin	Rural	1,540	74.9
	Urban	516	25.1
Monthly living expenses	<1,000 yuan	302	14.7
	1,000–1,499 yuan	1,129	54.9
	1,500–1999 yuan	484	23.5
	≥2000 yuan	141	6.9
Grade	Year 1	1,279	62.2
	Year 2	602	29.3
	Year 3	124	6.0
	Year 4	32	1.6
	Year 5	19	0.9
BMI category	<18.5 kg/m^2^	414	20.1
	18.5–<24.0 kg/m^2^	1,230	59.8
	24.0–<28.0 kg/m^2^	292	14.2
	≧28.0 kg/m^2^	120	5.8
Institution type	Medical	819	39.9
	Non-medical	1,234	60.1

BMI categories follow the Chinese adult standard WS/T 428–2013. Medical institutions include Gannan Medical University and one entry recorded as “medical junior college”; recognizable other schools are categorized as non-medical. Institution-type percentages use 2,053 classifiable records as the denominator (819/2,053 and 1,234/2,053); three school entries cannot be classified from their recorded text. This descriptive grouping does not alter the detailed school-group covariate in Table 2.

The 2,056 participants comprised 1,068 men (51.9%) and 988 women (48.1%). Mean age was 18.92 ± 1.19 years (range 18–29); mean height was 166.64 ± 8.54 cm, weight 59.88 ± 11.90 kg, and BMI 21.51 ± 3.75 kg/m^2^. Students from rural areas accounted for 74.9%; 90.7% were not only children. Monthly living expenses were most often 1,000–1,499 yuan (54.9%). Grade, reported school type, and BMI categories are provided in Table 4.

### 3.2 Descriptive statistics and correlations

Descriptive statistics and correlations among the main variables are presented in Table 5.

**Table 5:** Descriptive statistics and correlations among the main variables.

Variable	Mean	SD	1	2	3	4	5
1. Physical activity	14.43	17.96	1				
2. Negative fat talk	10.34	5.94	0.076^**^	1			
3.Wellbeing	15.94	5.84	0.040	−0.263^***^	1		
4. Loneliness	16.25	4.58	−0.066^**^	0.233^***^	−0.453^***^	1	
5. Sleep quality	5.87	3.58	−0.047^*^	0.204^***^	−0.494^***^	0.441^***^	1

Higher sleep quality scores indicate poorer sleep quality. ^*^p < 0.05; ^**^p < 0.01; ^***^p < 0.001.

The mean scores were 10.34 ± 5.94 for negative fat talk, 15.94 ± 5.84 for wellbeing, 16.25 ± 4.58 for loneliness, 14.43 ± 17.96 for physical activity, and 5.87 ± 3.58 for sleep quality. Of the 2,056 participants, 984 (47.9%) were classified as having poor sleep quality. Sleep quality was positively correlated with negative fat talk (*r* = 0.204, *p* < 0.001) and loneliness (*r* = 0.441, *p* < 0.001), and negatively correlated with wellbeing (*r* = −0.494, *p* < 0.001) and physical activity (*r* = −0.047, *p* = 0.038).

### 3.3 Multiple linear regression analysis

The results of the multiple linear regression analysis are shown in Table 1.

Multiple linear regression showed that, before adjustment for covariates, negative fat talk, wellbeing, and loneliness were significant predictors of sleep quality, whereas physical activity was not. After adjustment for sex, negative fat talk (*β* = 0.060, *p* = 0.003), wellbeing (*β* = −0.352, *p* < 0.001), and loneliness (*β* = 0.254, *p* < 0.001) remained significant predictors of sleep quality, whereas physical activity remained non-significant (*β* = 0.021, *p* = 0.301). The newly added fully adjusted model yielded *B* = 0.046 for negative fat talk (95% HC3 CI 0.016–0.076; standardized *β* = 0.076; *p* = 0.003; model *R*^2^ = 0.316), with similar directions for the other psychological variables (Table 2). Excluding four records with implausible BMI or out-of-range physical activity retained the association for negative fat talk (*B* = 0.047; 95% CI 0.017–0.078; *n* = 1966).

The final thesis reported confirmatory-factor fit values of CFI = 0.874, TLI = 0.824, RMSEA = 0.152 for the ULS-8 and CFI = 0.928, TLI = 0.892, RMSEA = 0.110 for PSQI. The original structural model fit was CFI = 0.890, TLI = 0.878, RMSEA = 0.073, and SRMR = 0.100. The unrotated first component in the additional 25-indicator diagnostic accounted for 31.84% of variance among 1956 indicator-complete records; this diagnostic alone cannot rule out shared-method bias.

### 3.4 Mediation analysis

The mediation effects are presented in Table 3.

The original latent-variable model reported a direct association of 0.057 and a total indirect association of 0.223, comprising 0.118 through wellbeing and 0.105 through loneliness (Table 3; Figure 1). The fully adjusted observed-score sensitivity model yielded a total association *B* = 0.151 (95% percentile-bootstrap CI 0.117–0.182), total indirect association *B* = 0.105 (CI 0.087–0.122), comprising 0.061 through wellbeing (CI 0.048–0.075) and 0.043 through loneliness (CI 0.033–0.055); its direct association was *B* = 0.046 (CI 0.015–0.076). The exploratory difference between the two indirect associations was *B* = 0.018 (CI −0.0005–0.034), giving an imprecise estimate of their relative magnitude. Latent-variable and observed-score coefficients are on different scales and were interpreted separately.

**Figure 1:**
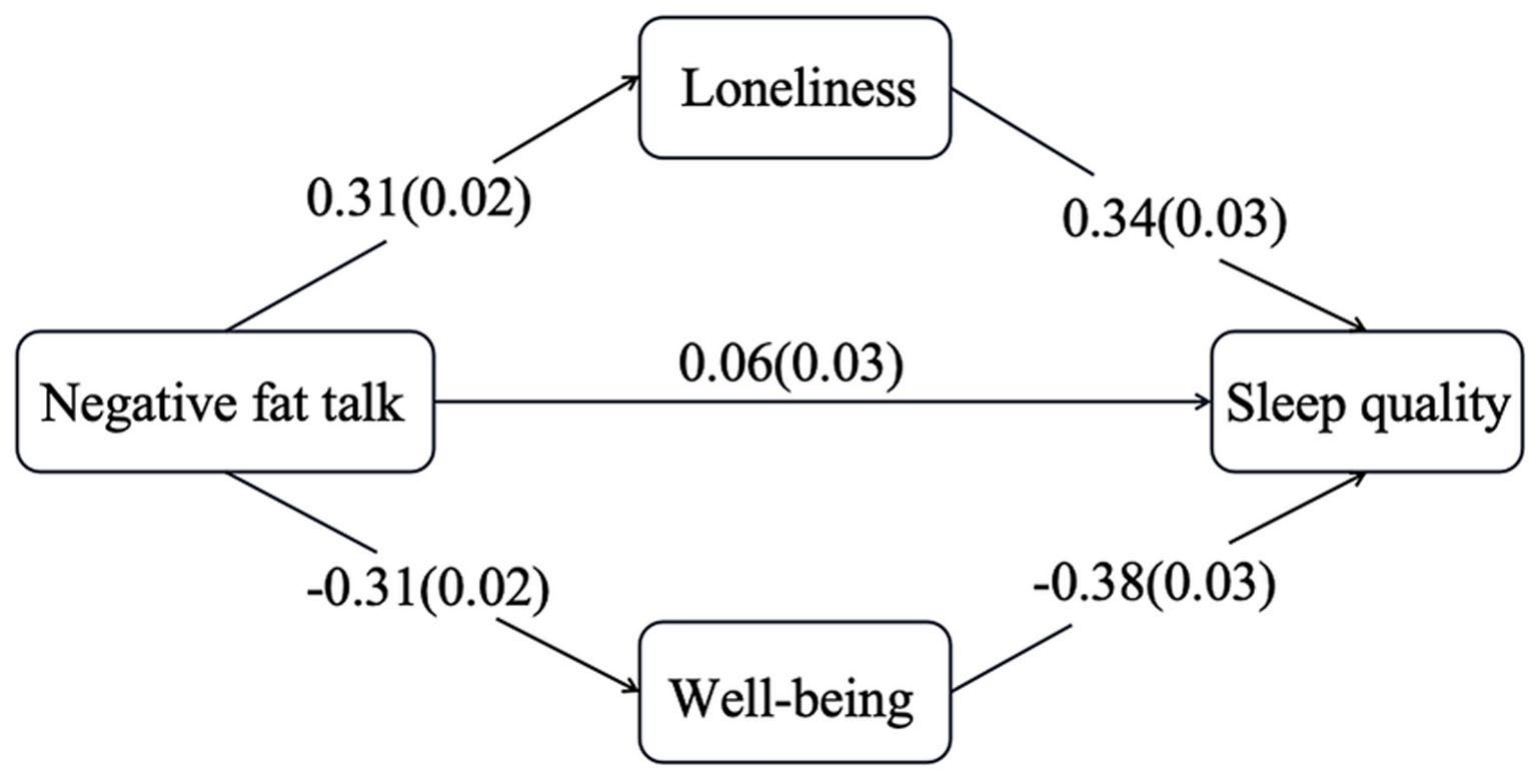
Parallel mediation model. Standardized path coefficients (standard errors) are shown.

The parallel mediation model is shown in Figure 1.

## 4 Discussion

### 4.1 Key findings and overall interpretation

Negative fat talk was associated with higher PSQI scores in both the original sex-adjusted regression and an additional model that included age, BMI, physical activity, socioeconomic and academic variables, and school group. Wellbeing and loneliness accounted for parallel indirect associations in the original latent model and the adjusted observed-score sensitivity analysis. These results characterize associations among concurrently measured variables.

The findings support a sleep health perspective in which sleep quality is considered alongside body image, positive psychological resources, and social connectedness. For students in university settings, body-related negative self-expression may provide a visible entry point for recognizing psychosocial vulnerability related to sleep health. This interpretation is consistent with body image research showing that sociocultural pressures, appearance internalization, and body-related self-evaluation are closely linked to psychological and behavioral outcomes (Kidd et al., 2024; Rodgers et al., 2014; Schaefer et al., 2015).

This finding is broadly consistent with previous research. For example, a cross-sectional study of 1,258 university students in southern China found that body dissatisfaction was significantly associated with poorer sleep quality and higher levels of depression (Hao et al., 2023). Another study of young women with disordered eating symptoms reported that greater weight bias internalization was associated with more severe insomnia symptoms (Christensen Pacella and Forbush, 2024). Together, these findings indicate a stable link between body-related evaluative pressure and impaired nocturnal recovery. The present study extends this evidence to Chinese college students and suggests that negative body-related expression may be relevant to sleep health in this population.

At the same time, this study advances existing knowledge by moving beyond separate discussions of body dissatisfaction and sleep, wellbeing and sleep, or loneliness and sleep. A systematic review on positive affect and sleep showed that positive affect is generally accompanied by better subjective sleep experiences (Ong et al., 2017), and a study of university students in southern China found that loneliness, bedtime procrastination, and rumination jointly predicted sleep quality (Wang et al., 2025). However, these studies did not place negative fat talk, wellbeing, loneliness, and sleep within one mediation framework. Our findings indicate that negative fat talk is associated with sleep quality through two intertwined pathways involving positive psychological resources and adverse social-psychological experience. The contribution of this study lies in identifying negative fat talk as a body-related psychosocial factor of poor sleep quality and in showing that wellbeing and loneliness represent two parallel dimensions of student sleep health: positive psychological resources and perceived social connectedness.

The study brings negative fat talk, wellbeing, loneliness, and sleep quality into one framework. The larger adjusted observed-score indirect estimate for wellbeing was accompanied by a contrast confidence interval that crossed zero, supporting a cautious interpretation of relative strength. Evidence relating loneliness to psychological and subjective wellbeing among university students (Çiçek, 2021), and recent work on social media, self-esteem, and loneliness (Ünsal et al., 2025), provide context for the intertwined psychological and interpersonal correlates in this study.

### 4.2 Interpretation of the mechanisms

From an intrapersonal perspective, negative fat talk may reflect students’ repeated self-monitoring, social comparison and body-related shame in daily interactions. The expression may further strengthen the individual’s concern for their own physical defects, sensitivity to external evaluation, and internalization of appearance ideals. These psychological processes are closely related to ruminative thinking and cognitive arousal before bedtime, and the latter are closely related to the start and maintenance of sleep (Kidd et al., 2024; Warren et al., 2012).

From the perspective of positive psychological resources, wellbeing may constitute an important protective factor for college students’ sleep health. Existing studies show that the sense of vitality, relaxation and interest in daily life captured by wellbeing are closely related to sleep status. A meta-analysis of longitudinal study of adolescents in 2024 found that there is a continuous interaction between sleep, mental health and positive wellbeing (Bacaro et al., 2024). Another systematic review of positive emotions and sleep shows that higher levels of positive emotions are usually associated with better sleep (Ong et al., 2017). Against this background, continuous negative fat talk may weaken the individual’s positive experience and recovery ability, allowing reduced daytime psychological energy to spill over into the night, thus increasing the possibility of sleep being disturbed. Therefore, the mediating role of wellbeing suggests that the decline in sleep quality is not only related to the increase in negative emotions, but also to the loss of positive psychological resources.

From the perspective of social psychology, loneliness may reflect an important dimension of the lack of social connection in college students’ sleep health. Negative fat talk is often accompanied by experiences such as peer comparison, fear of evaluation and social withdrawal. In the college environment, these experiences may weaken the individual’s sense of belonging and increase social vigilance. A longitudinal analysis published by HUNT Research in Trøndelag, Norway in 2026 shows that there is a two-way relationship between loneliness and sleep disorders in the development from adolescence to middle age (Parlikar et al., 2026). Similarly, a study of university students in southern China showed that greater loneliness was associated with poorer sleep quality (Wang et al., 2025). The present findings suggest that loneliness is one pathway through which negative body-related expression is associated with poor sleep quality.

Taken together, wellbeing and loneliness represent two complementary psychosocial dimensions of student sleep health: internal restorative resources and perceived social connectedness. Their parallel roles help explain why negative fat talk, although expressed in everyday language, may be associated with sleep quality through psychological and social pathways.

Body ideals and body talk can differ by sex: thinness-focused concerns may be especially salient for some women, whereas muscularity-related concerns may be more salient for some men (Engeln et al., 2013; Lin et al., 2021). The current measure covered negative fat talk only, and the main models pooled all participants. Future research could test measurement invariance and sex moderation with measures of both thinness- and muscularity-focused talk.

### 4.3 Further discussion of complex findings

The attenuated direct association after including wellbeing and loneliness suggests that the relationship between negative fat talk and sleep quality may operate mainly through psychosocial pathways. The findings highlight the importance of examining intermediate psychological and social factors when understanding the poor sleep quality of college students.

There is only a weak two-variable correlation between physical activity and sleep quality, and its role in the adjusted model is relatively limited. This result may be related to the way physical activity is measured. The measurement of physical activity in this study mainly covers intensity, frequency and duration, while the reflection of information on activity type, activity time and social situation is relatively limited. Different forms of physical activity may differ in their emotional restorative and socially connective functions. A meta-analytic review shows that physical activity is generally associated with better sleep (Kredlow et al., 2015). Therefore, it is necessary for future research to adopt more refined measurement tools to further clarify the role of physical activity in the sleep health of college students.

### 4.4 Limitations

When explaining the results of the mediation effect of this study, several limitations need to be considered. First, the convenience sample was recruited through classroom QR codes and teacher-forwarded links across the October 2024–July 2025 period. Response dates and verified recruitment denominators were unavailable, so temporal differences and participation selection remain possible. The free-text school variable contains spelling variants and additional named institutions beyond the four sites described in the final thesis; school-specific representativeness requires verified recruitment records.

Second, the study adopts a cross-sectional research design. Although the path relationship between variables has been tested using the structural equation model, the causal order between variables cannot be determined yet. Future research can adopt causal direction of the intermediary path and examine the dynamic process of changing the relationship between variables over time.

Third, all core variables were assessed by self-report, which may have introduced common method bias and social desirability bias (Podsakoff et al., 2003). This should be taken into account when interpreting the observed associations.

In addition, a number of statistical and surveying issues need to be considered. The analysis of this study is based on the existing data set, and further sensitivity analysis help to enhance the robustness of the research results. Some measurement model indicators are lower than the usually recommended evaluation criteria, so the results of the mediation findings are more suitable to be interpreted as a correlation between the scores of each scale. The measurement of physical activity only covers limited information, and the assessment of activity type, activity time and social situation is relatively insufficient, which may reduce its explanatory effectiveness.

### 4.5 Practical implications and future research

Research results show that the work of promoting sleep health in colleges and universities is expected to achieve better results through the integration of body image education, media literacy cultivation, emotional resource construction and peer support strategies. In a non-clinical college environment, negative fat talk may provide a more intuitive entry point for identifying students who experience appearance-related pressure and sleep-related social and psychosocial vulnerabilities. College health services can integrate positive body communication and integrate social connectedness into sleep health education. Future research can use longitudinal research, diary research or ecological momentary assessment to further examine the relevant action path, and optimize and improve the measurement methods of variables such as negative fat talk, sleep, and physical activity.

## 5 Conclusion

In the sample of Chinese college students included in this study, poor sleep quality was common. Negative fat talk is significantly associated with poor sleep quality, which can be statistically explained by lower wellbeing and higher loneliness. The research results show that body-related self-expression, psychological resources and social connection are important socio-psychological factors closely related to the promotion of sleep health in colleges and universities.

## Data Availability

The raw data supporting the conclusions of this article will be made available by the authors, without undue reservation.

## References

[ref1] Akram U. Drabble J. Irvine K. Allen S. Akram A. Stevenson J. C. . (2025). Prevalence and psychiatric correlates of loneliness in UK university students. Npj Ment. Health Res. 4:45. doi: 10.1038/s44184-025-00144-8, 40957876 PMC12441134

[ref2] Bacaro V. Miletic K. Crocetti E. (2024). A meta-analysis of longitudinal studies on the interplay between sleep, mental health, and positive wellbeing in adolescents. Int. J. Clin. Health Psychol. 24:100424. doi: 10.1016/j.ijchp.2023.100424, 38125984 PMC10730350

[ref3] Barbeau K. Carbonneau N. Pelletier L. (2022). Family members and peers’ negative and positive body talk: how they relate to adolescent girls’ body talk and eating disorder attitudes. Body Image 40, 213–224. doi: 10.1016/j.bodyim.2021.12.010 35026685

[ref4] Brown Z. Tiggemann M. (2016). Attractive celebrity and peer images on Instagram: effect on women’s mood and body image. Body Image 19, 37–43. doi: 10.1016/j.bodyim.2016.08.007 27598763

[ref5] Buysse D. J. Reynolds C. F. 3rd Monk T. H. Berman S. R. Kupfer D. J. (1989). The Pittsburgh sleep quality index: a new instrument for psychiatric practice and research. Psychiatry Res. 28, 193–213. doi: 10.1016/0165-1781(89)90047-4, 2748771

[ref6] Cash T. F. (2004). Body image: past, present, and future. Body Image 1, 1–5. doi: 10.1016/s1740-1445(03)00011-1, 18089136

[ref7] Christensen Pacella K. A. Forbush K. T. (2024). Weight bias internalization is positively associated with insomnia symptom severity in young women with disordered eating. Sleep Health 10, 60–64. doi: 10.1016/j.sleh.2023.10.014, 38016909 PMC10922884

[ref8] Çiçek İ. (2021). Mediating role of self-esteem in the association between loneliness and psychological and subjective wellbeing in university students. Int. J. Contemp. Educ. Res. 8, 83–97. doi: 10.33200/ijcer.817660

[ref9] Deng M. Qian M. Lv J. Guo C. Yu M. (2023). The association between loneliness and sleep quality among older adults: a systematic review and meta-analysis. Geriatr. Nurs. 49, 94–100. doi: 10.1016/j.gerinurse.2022.11.013, 36470104

[ref10] Engeln R. Sladek M. R. Waldron H. (2013). Body talk among college men: content, correlates, and effects. Body Image 10, 300–308. doi: 10.1016/j.bodyim.2013.02.001, 23490553

[ref11] Fardouly J. Diedrichs P. C. Vartanian L. R. Halliwell E. (2015). Social comparisons on social media: the impact of facebook on young women’s body image concerns and mood. Body Image 13, 38–45. doi: 10.1016/j.bodyim.2014.12.002 25615425

[ref12] Faul F. Erdfelder E. Buchner A. Lang A. G. (2009). Statistical power analyses using G*Power 3.1: tests for correlation and regression analyses. Behav. Res. Methods 41, 1149–1160. doi: 10.3758/brm.41.4.1149, 19897823

[ref13] Festinger L. (1954). A theory of social comparison processes. Hum. Relat. 7, 117–140. doi: 10.1177/001872675400700202

[ref14] Fredrickson B. L. Roberts T.-A. (1997). Objectification theory: toward understanding women’s lived experiences and mental health risks. Psychol. Women Q. 21, 173–206. doi: 10.1111/j.1471-6402.1997.tb00108.x

[ref15] Hao M. Liu X. Wang Y. Wu Q. Yan W. Hao Y. (2023). The associations between body dissatisfaction, exercise intensity, sleep quality, and depression in university students in southern China. Front. Psych. 14:1118855. doi: 10.3389/fpsyt.2023.1118855, 37020733 PMC10067572

[ref16] Hays R. D. DiMatteo M. R. (1987). A short-form measure of loneliness. J. Pers. Assess. 51, 69–81. doi: 10.1207/s15327752jpa5101_6, 3572711

[ref17] Hooper L. Puhl R. Eisenberg M. E. Crow S. Neumark-Sztainer D. (2021). Weight teasing experienced during adolescence and young adulthood: cross-sectional and longitudinal associations with disordered eating behaviors in an ethnically/racially and socioeconomically diverse sample. Int. J. Eat. Disord. 54, 1449–1462. doi: 10.1002/eat.23534, 33969902 PMC8355094

[ref18] Hu L. Bentler P. M. (1999). Cutoff criteria for fit indexes in covariance structure analysis: conventional criteria versus new alternatives. Struct. Equ. Model. 6, 1–55. doi: 10.1080/10705519909540118

[ref19] Kidd C. Loxton N. J. Uhlmann L. R. Donovan C. L. (2024). Integrating social media, body shame and psychological distress within the elaborated sociocultural model. Body Image 50:101723. doi: 10.1016/j.bodyim.2024.101723, 38788591

[ref20] Kredlow M. A. Capozzoli M. C. Hearon B. A. Calkins A. W. Otto M. W. (2015). The effects of physical activity on sleep: a meta-analytic review. J. Behav. Med. 38, 427–449. doi: 10.1007/s10865-015-9617-6 25596964

[ref21] Li L. Wang Y. Y. Wang S. B. Zhang L. Li L. Xu D. D. . (2018). Prevalence of sleep disturbances in Chinese university students: a comprehensive meta-analysis. J. Sleep Res. 27:e12648. doi: 10.1111/jsr.12648, 29383787

[ref22] Lin L. Flynn M. O’Dell D. (2021). Measuring positive and negative body talk in men and women: the development and validation of the body talk scale. Body Image 37, 106–116. doi: 10.1016/j.bodyim.2021.01.013, 33610049

[ref23] Lin J. Wang D. Wang W. Chen H. Lin W. Yin L. . (2025). Concurrent and longitudinal associations of dissatisfaction with height and weight, body mass index with sleep disturbance among adolescents in China. Eur. Child Adolesc. Psychiatry 34, 341–350. doi: 10.1007/s00787-024-02499-3, 38967648

[ref24] Mills J. Fuller-Tyszkiewicz M. (2016). Fat talk and its relationship with body image disturbance. Body Image 18, 61–64. doi: 10.1016/j.bodyim.2016.05.001, 27286565

[ref25] National Health and Family Planning Commission of the PRC (2013). Adult Weight Determination. Beijing: China Standards Press.

[ref26] Ong A. D. Kim S. Young S. Steptoe A. (2017). Positive affect and sleep: a systematic review. Sleep Med. Rev. 35, 21–32. doi: 10.1016/j.smrv.2016.07.006, 27553441

[ref27] Ostrow K. D. Rieur O. Moeller R. W. Seehuus M. (2025). From sleeplessness to solitude: emotional repair as a buffer between insomnia and loneliness in university students. Front. Sleep 4:1516094. doi: 10.3389/frsle.2025.1516094, 40134810 PMC11936514

[ref28] Palmer C. A. Bower J. L. Cho K. W. Clementi M. A. Lau S. Oosterhoff B. . (2024). Sleep loss and emotion: a systematic review and meta-analysis of over 50 years of experimental research. Psychol. Bull. 150, 440–463. doi: 10.1037/bul0000410, 38127505

[ref29] Parlikar N. Power J. M. Hyland P. Coogan A. N. Kvaløy K. Strand L. B. . (2026). Reciprocal effects between loneliness and sleep disturbances from adolescence to mid-adulthood: the hunt study. Sleep Adv. 7:zpag004. doi: 10.1093/sleepadvances/zpag004, 41768373 PMC12936869

[ref30] Podsakoff P. M. MacKenzie S. B. Lee J. Y. Podsakoff N. P. (2003). Common method biases in behavioral research: a critical review of the literature and recommended remedies. J. Appl. Psychol. 88, 879–903. doi: 10.1037/0021-9010.88.5.879, 14516251

[ref31] Richard A. Rohrmann S. Lohse T. Eichholzer M. (2016). Is body weight dissatisfaction a predictor of depression independent of body mass index, sex and age? Results of a cross-sectional study. BMC Public Health 16:863. doi: 10.1186/s12889-016-3497-8, 27558007 PMC4995657

[ref32] Rodgers R. F. Paxton S. J. McLean S. A. (2014). A biopsychosocial model of body image concerns and disordered eating in early adolescent girls. J. Youth Adolesc. 43, 814–823. doi: 10.1007/s10964-013-0013-7, 24014348

[ref33] Schaefer L. M. Burke N. L. Thompson J. K. Dedrick R. F. Heinberg L. J. Calogero R. M. . (2015). Development and validation of the sociocultural attitudes towards appearance questionnaire-4 (sataq-4). Psychol. Assess. 27, 54–67. doi: 10.1037/a0037917, 25285718

[ref34] Scott A. J. Webb T. L. Martyn-St James M. Rowse G. Weich S. (2021). Improving sleep quality leads to better mental health: a meta-analysis of randomised controlled trials. Sleep Med. Rev. 60:101556. doi: 10.1016/j.smrv.2021.101556, 34607184 PMC8651630

[ref35] Seoane H. A. Moschetto L. Orliacq F. Orliacq J. Serrano E. Cazenave M. I. . (2020). Sleep disruption in medicine students and its relationship with impaired academic performance: a systematic review and meta-analysis. Sleep Med. Rev. 53:101333. doi: 10.1016/j.smrv.2020.101333, 32485517

[ref36] Topp C. W. Østergaard S. D. Søndergaard S. Bech P. (2015). The who-5 wellbeing index: a systematic review of the literature. Psychother. Psychosom. 84, 167–176. doi: 10.1159/000376585, 25831962

[ref37] Ünsal F. Korkmaz Z. Çiçek İ. Abdullah Alshehri N. Mohammed Abdullah Alkhulayfi A. Yıldırım M. (2025). Mediating roles of self-esteem and positive childhood experiences in the relationship between problematic social media use and loneliness. Psicol. Reflex. Crit. 38:28. doi: 10.1186/s41155-025-00364-z, 41082046 PMC12518197

[ref38] Wang Y. Wang X. Wang Q. Liu G. Wu C. Hao M. (2025). The role of bedtime procrastination, rumination, loneliness, and positive body image in predicting sleep quality among university students: a sex-specific analysis. Alpha Psychiatry 26:44142. doi: 10.31083/ap44142, 40630886 PMC12231379

[ref39] Warren C. S. Holland S. Billings H. Parker A. (2012). The relationships between fat talk, body dissatisfaction, and drive for thinness: perceived stress as a moderator. Body Image 9, 358–364. doi: 10.1016/j.bodyim.2012.03.008, 22521180

[ref40] Wenig V. Heinrichs K. Heumann E. Lehnchen J. Burian J. Deptolla Z. . (2025). Social-ecological factors associated with loneliness in university students: results from the German cross-sectional Studibifra study. Front. Psych. 16:1469811. doi: 10.3389/fpsyt.2025.1469811, 40692650 PMC12277272

[ref41] Wu Y. Tang J. Meng X. Wang F. Sun X. Fan S. . (2024). Validation and structural exploration of the Chinese version of the body talk scale. Body Image 51:101766. doi: 10.1016/j.bodyim.2024.101766, 38986235

[ref42] Yang F. Qi L. Liu S. Hu W. Cao Q. Liu Y. . (2022). Body dissatisfaction and disordered eating behaviors: the mediation role of smartphone addiction and depression. Nutrients 14:1281. doi: 10.3390/nu14061281, 35334936 PMC8955505

[ref43] Ybarra O. Chan T. (2025). Personal agency and social connection are associated with loneliness over time. Commun. Psychol. 3:151. doi: 10.1038/s44271-025-00329-z, 41162636 PMC12572240

[ref44] Yuan S. You M. (2022). Effects of physical activity on college students’ subjective wellbeing during Covid-19. J. Epidemiol. Glob. Health 12, 441–448. doi: 10.1007/s44197-022-00062-4, 36197597 PMC9533984

[ref45] Zheng Y. Qualter P. Rollano C. Hu X. Martin A. Nukui M. . (2026). Impact of loneliness on health in healthy populations: a meta-analysis. Br. J. Health Psychol. 31:e70040. doi: 10.1111/bjhp.70040, 41355074 PMC12683082

